# Vibrationally Mode-Specific Molecular Energy Transfer to Surface Electrons in Metastable Formaldehyde Scattering from Cesium-Covered Au(111)

**DOI:** 10.1021/acs.jpca.4c02184

**Published:** 2024-06-08

**Authors:** Behrouz Sabour, Roman J. V. Wagner, Bastian C. Krüger, Alexander Kandratsenka, Alec M. Wodtke, Tim Schäfer, G. Barratt Park

**Affiliations:** † Department of Chemistry and Biochemistry, Texas Tech University, Box 41061 Lubbock, Texas 79409-1061, United States; ‡ Max-Planck-Institut für Multidisziplinäre Naturwissenschaften, Am Faßberg 11, Göttingen 37077, Germany; § Georg-August-Universität Göttingen, Institut für physikalische Chemie, Tammanstr. 6, Göttingen 37077, Germany; ∥ International Center for Advanced Studies of Energy Conversion, University of Göttingen, Göttingen 37077, Germany

## Abstract

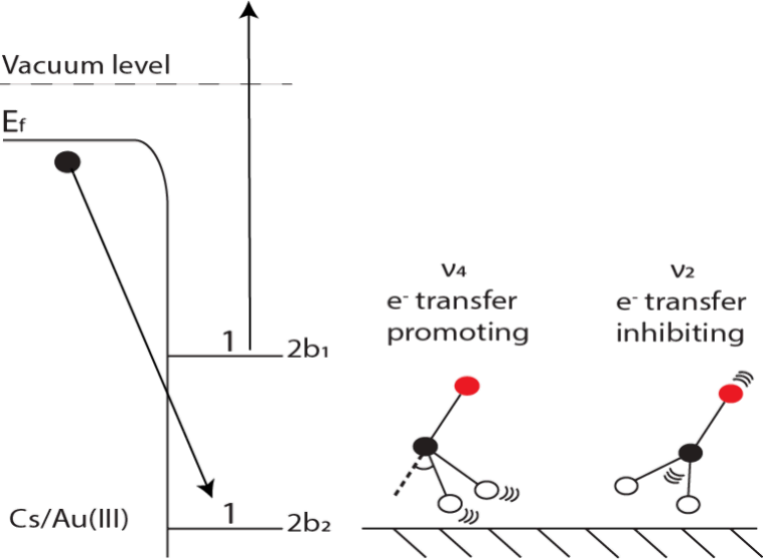

Nonadiabatic interaction of adsorbate nuclear motion with the continuum of electronic states is known to affect the dynamics of chemical reactions at metal surfaces. A large body of work has probed the fundamental mechanisms of such interactions for atomic and diatomic molecules at surfaces. In polyatomic molecules, the possibility of mode-specific damping of vibrational motion due to the effects of electronic friction raises the question of whether such interactions could profoundly affect the outcome of chemistry at surfaces by selectively removing energy from a particular intramolecular adsorbate mode. However, to date, there have not been any fundamental experiments demonstrating nonadiabatic electron-vibration coupling in a polyatomic molecule at a surface. In this work, we scatter excited metastable formaldehyde and formaldehyde-d_2_ from a low work function surface and detect ejected exoelectrons that accompany molecular relaxation. The exoelectron ejection efficiency exhibits a strong dependence on the vibrational mode that is excited: out-of-plane bending excitation (ν_4_) leads to significantly more exoelectrons than does CO stretching excitation (ν_2_). The results provide clear evidence for mode-specific energy transfer from vibration to surface electrons.

## Introduction

Molecular dynamics at metal surfaces play a central role in heterogeneous catalysis. Electronically adiabatic potential energy surfaces are commonly used to understand the motion of chemical reactants through transition states, although it has been recognized that such treatments fail in cases where the coupling of surface adsorbate motion to electronic degrees of freedom is significant.^1,2^ There are a number of well-studied examples of such systems. The sticking of hyperthermal hydrogen atoms at metal surfaces is dominated by electronically nonadiabatic dissipation of translational energy to electron–hole pairs.^3−7^ Diatomic molecules are known to undergo vibrational excitation^8−14^ or relaxation^15−21^ as a result of energy exchange with surface electrons. Exothermic chemical reactions can also excite electron–hole pairs, leading to macroscopic “chemicurrents” that can be observed during adsorption and recombination reactions at the surface of a metal–insulator–metal (MIM) diode.^22^

A particularly striking example of electronically nonadiabatic energy exchange at surfaces has been observed during the scattering of highly vibrationally excited NO molecules from a low work function surface generated by depositing submonolayer coverages of Cs on Au(111).^23^ When the vibrational energy exceeds the surface work function, surface exoelectrons are ejected from the vacuum. This phenomenon can be described only by a strong coupling mechanism in which the adsorbate imparts sufficient energy to a single electron to overcome the surface work function. The measured incidence velocity dependence^24^ and exoelectron kinetic energy distribution^25^ are indicative of a vibrational autodetachment mechanism involving transient formation of the NO^–^ anion at distances of ∼10 Å from the surface.

Vibrational enhancement of exoelectron efficiencies has also been observed when scattering molecules in metastable electronically excited states from metal surfaces.^26−28^ Exoelectrons are detected when a Stark decelerated beam of CO molecules in the vibrationless *a*^3^Π(*v* = 0) metastable state is scattered from a Au(111) surface—the exoelectron efficiency is enhanced by a factor of ∼2.6 when the molecules are excited to *a*^3^Π(*v* ≥ 4) levels using a Franck–Condon pumping scheme.^28^ The mechanism is believed to involve resonant transfer of an electron near the Fermi level, leading to transient formation of CO^–^ anion at stretched CO bond lengths and at distances of ca. 5.0–5.4 Å from the surface, followed by vibrational autodetachment. Vibrational enhancement can be explained by vibrational excitation causing the molecule to spend more time near the outer vibrational turning point, which increases the lifetime of the resonance.

Nonadiabatic interaction of adsorbate motion with surface electron hole pairs poses significant challenges for theory. Although the dynamics of hydrogen atoms at metal surfaces has been well described using electronic friction in the local density friction approximation,^29,30^ there are no adequate theories presently available to describe hydrogen atom scattering from semiconductors, in which electron–hole pair excitations across the bandgap are possible.^31^ Electronic friction using time-dependent perturbation theory on Kohn–Sham orbitals has succeeded in describing mode-specific quenching of diatomic adsorbate surface modes,^32^ but strong coupling mode-specific phenomena such as those described in this work require more explicit theories. Success has been achieved in explaining vibrational excitation via electron transfer mechanisms using the independent electron surface hopping theory,^33^ but this theory fails to predict qualitative trends in the incidence energy dependence of multiquantum vibrational energy loss in NO on Au(111),^34^ possibly due to problems in the underlying density functional theory based potential energy surfaces.^35^ Such failures highlight the need for fundamental experiments to serve as benchmarks for nascent theoretical approaches.

So far, detailed mechanistic studies have focused almost exclusively on atoms or diatomic molecules at surfaces. Lifetimes of various metal–adsorbate vibrational modes indicate that electronically nonadiabatic energy exchange mechanisms can be strongly mode dependent. For example, for CO adsorbed on Cu(100), the C–O stretching mode has a much shorter lifetime (2 ps) than the frustrated translational mode (40 ps).^15,17,36^ This raises fundamental questions as to whether mode-selective damping of different *intramolecular* adsorbate modes might influence the outcome of polyatomic molecule chemistry at metal surfaces. Time-dependent perturbation theory indicates that electron–hole pair induced relaxation rates in rhenium complexes on Au(111) are strongly mode dependent,^37^ suggesting that mode-selective damping might occur commonly at metal surfaces. However, to date, there has been no direct demonstration of nonadiabatic energy exchange mechanisms between polyatomic molecular vibrations and metal surface electrons: in the scattering of ammonia^38^ and acetylene^39^ from Au(111), vibrational excitation occurs via translation-to-vibration (T-V) coupling, whereas in the scattering of vibrationally excited methane from Ni(111)^40^ and Au(111),^41^ energy transfer is understood to occur through an electronically adiabatic surface-induced intramolecular vibrational energy redistribution (IVR) mechanism.

This paper describes the surface scattering of formaldehyde (H_2_CO). For decades, formaldehyde has served the molecular physics community as an important prototype that is “just large enough” to reveal fundamental complexities that arise in the spectroscopy^42^ and photochemical dynamics^43,44^ of polyatomic molecular systems. The weak, spin-forbidden (singlet-to-triplet) *ã* ^3^A_2_ ← *X̃* ^1^A_1_ transition starting at 397 nm is associated with a (2*b*_2_)^1^(2*b*_1_)^1^ ← (2*b*_2_)^2^ electronic valence orbital promotion from a nonbonding oxygen 2p_*y*_ orbital to an antibonding CO π* orbital. The ground *X̃* state has a planar geometry, whereas the electronically excited *ã* state suffers from a vibronic interaction that causes the H atoms to bend 40° out of plane and gives rise to a double-minimum potential energy surface with an inversion barrier of 776 cm^–1^.^45^ The *ã* ← *X̃* transition borrows intensity through a spin–orbit perturbation. The vibrational structure is dominated by Franck–Condon progressions in the totally symmetric (a_1_) combinations of the ν_2_ (CO stretch, a_1_) and ν_4_ (out-of-plane bending, b_1_) modes. Throughout this paper, we denote the number of vibrational quanta in the upper (*v*′) and lower (*v*″) states using the notation .

In this work, we report mode-dependent exoelectron generation efficiencies in the scattering of vibrationally excited formaldehyde in the metastable *ã* ^3^A_2_ electronic state from thin films of Cs supported on a Au(111) substrate. To the best of our knowledge, this is the first clear experimental demonstration of a mode-specific energy exchange mechanism between a polyatomic molecular vibration and surface electronic degrees of freedom.

## Methods

Experiments were performed in a differentially pumped molecular beam/UHV surface science apparatus used previously for surface scattering experiments on formaldehyde.^46,47^ A molecular beam of formaldehyde was generated by cracking solid paraformaldehyde, 97% purity, (or paraformaldehyde-d_2_, 99 atom % D) at 85 °C in a heated sample holder that is built into a home-built pulsed solenoid valve, as described in ref (48). The molecular beam is seeded in He at a backing pressure of 10.8 bar, which results in a mean speed of 1700 m/s and a rotational temperature of 4–10 K.

A portion of the incident formaldehyde beam is excited to specific vibrational levels of the *ã* ^3^A_2_ electronic state by directly pumping the rovibrationally resolved, spin-forbidden *ã* ^3^A_2_ ← *X̃* ^1^A_1_ transition^49^ using a pulsed dye laser that intersects the molecular beam 8 mm upstream from the surface. The pulsed dye laser (Sirah Cobra Stretch SL, operating with Styryl 8 dye) was pumped at 10 Hz by the frequency-doubled output of a Nd:YAG laser (Continuum Powerlite 8010). The dye laser output was frequency doubled in a β-barium borate crystal (44° cut angle) to obtain tunable UV radiation with 10 ns pulse duration and typically 2–4 mJ pulse energy between 370 and 397 nm. The beam was collimated by a telescope to a diameter of ∼3 mm. A small portion of the dye laser fundamental was coupled to a high-precision wavemeter (High Finesse, WS7) for frequency calibration. Exoelectrons were collected by a set of ion optics located a short distance in front of the surface and sent onto a chevron configuration microchannel plate (MCP) detector.

A low work function surface was prepared in a UHV chamber (base pressure of 10^–10^ Torr) by depositing Cs on Au(111) using a commercially available source (SAES Getters). The effects of Cs dosing were monitored by observing the photoemission current generated by a He–Ne laser (*h*ν = 1.96 eV). See Figure S1. The photoemission current is known to reach a peak intensity at a coverage of 0.22–0.35 monolayers (ML)^50^ (at ∼4.1 min in Figure S1). Subsequently, the photoemission current goes through a minimum and then reaches a plateau after ∼7 min, which corresponds to an estimated dosage of ∼0.38–0.60 ML, with a work function of 1.6 eV.^51^ At this point, we stopped dosing and observed a photoemission current that is constant to within 15% over a period of 1 h. When we run the molecular beam under these conditions and pump the 4_0_^4^ transition, we obtain a stable exoelectron signal for at least 45 min after dosing. To ensure reproducible surface conditions, all exoelectron measurements were performed during a 30 min window after dosing. Subsequently, the Cs film was removed by sputtering with Ar^+^ for 20 min and annealing to 700 °C for an additional 20 min. Auger electron spectroscopy (AES) confirms that this regenerates the pristine Au(111) surface. The process is repeated, and the experiment is continued after another round of Cs dosing. All scattering experiments were performed at a surface temperature of 300 K.

## Results

**Figure 1:**
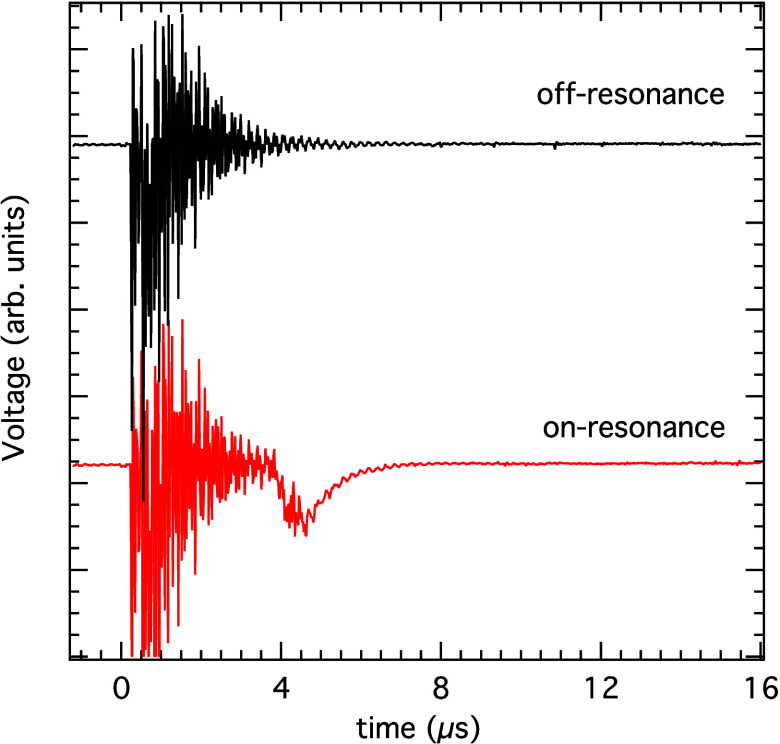
Voltage response versus time trace of the multichannel plate detector. Ringing is induced by scattered light when the laser is fired at 0 μs. When the laser is on-resonance with an *ã* ^3^*A*_2_ ← *X̃* ^1^*A*_1_ transition, a dip is observed due to the emission of exoelectrons after the ∼4 μs flight time of the laser-excited metastable molecules to the surface. The integrated dip intensity is proportional to the number of exoelectrons emitted.

**Figure 2:**
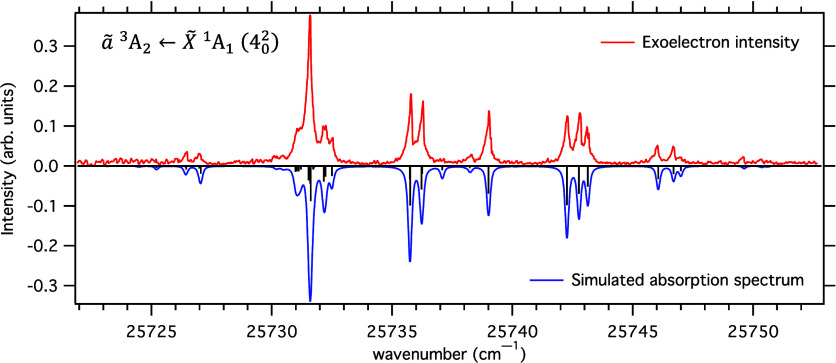
Exoelectron signal that is obtained as the laser is scanned through the *ã* ^3^*A*_2_ ← *X̃* ^1^*A*_1_ (4_0_^2^) transition is plotted as a function of the wavenumber of the laser (red, upward directed signal). The simulated *ã* ← *X̃* absorption intensities are shown for comparison (blue, downward directed signal). The black line spectrum shows the calculated positions and intensities of individual rotational and fine structure components. (See text for details of the simulation.)

The voltage response of the MCP detector is shown in Figure 1. Ringing on the detector arises from scattered laser light at 0 μs and decays after a few microseconds. After the ∼4 μs of flight time between the laser and the surface, the emission of exoelectrons gives rise to a dip in the voltage response. Exoelectron signals can only be seen when the laser is on resonance with a transition. Figure 2 shows the exoelectron signal obtained when the laser is scanned through the *ã* ^3^A_2_ ← *X̃* ^1^A_1_ (4_0_^2^) transition. The simulated absorption spectrum with rotational and fine structures is shown for comparison. Line positions were calculated using the spectroscopic constants of Birss et al.^52^ and the intensities of fine structure components were calculated according to the treatment described by Hougen.^53^

Spectra similar to the one shown in Figure 2 were obtained for the vibrationless 4_0_^0^ transition as well as for the four lowest-lying Franck–Condon bright vibronic transitions (4_0_^2^, 4_0_^4^, 2_0_^1^, and 2_0_^1^4_0_^2^). Experiments were also performed by pumping the same transitions in the D_2_CO isotopologue. Spectra from different bands were recorded back-to-back (in randomized order) and the measurements were repeated on several different days. The intensity was found to depend linearly on the laser power. We monitored the power continuously and applied a power correction. The lifetimes of rovibronic levels probed^54^ are significantly longer than the flight time to the surface, so no correction was made for decay of the metastable state.

**Figure 3:**
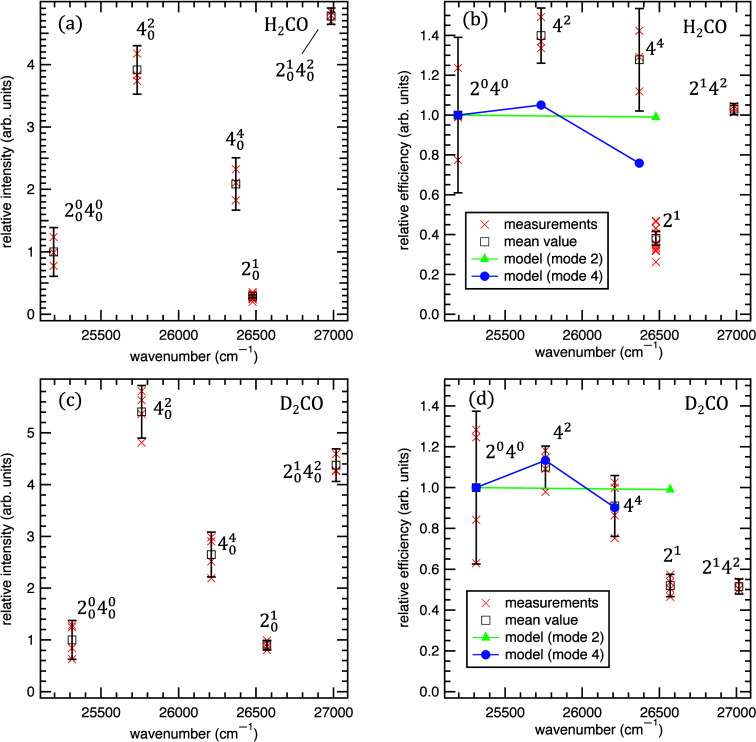
Exoelectron intensities and efficiencies for different *ã*-state vibrational levels, plotted as a function of excitation wavenumber. Panels (a) and (c) show the relative exoelectron intensities, corrected for laser power for the H_2_CO and D_2_CO isotopologues, respectively. Panels (b) and (d) show the efficiency, γ, obtained after correcting for the Franck–Condon factors, as described in the text for H_2_CO and D_2_CO, respectively. The results of individual measurements are shown as red crosses. The mean value obtained from each band is shown as an empty black box, and vertical error bars represent the 90% confidence interval obtained from repeated measurements. The prediction of a simple model for the 2^*n*^ (green triangles) and 4^*n*^ (blue circles) progression is shown in panels (b) and (d) for comparison (see text for details).

The integrated exoelectron signal intensities for each upper state, *v*′, are plotted as a function of excitation energy for H_2_CO and D_2_CO in Figures 3a and 3c, respectively. These intensities are given by
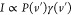
1where *P*(*v*′) is the population of the laser-excited metastable level, which is proportional to the Franck–Condon factor for the *ã* ^3^A_2_ ← *X̃* ^1^A_1_ (*v*′ ← *v*″ = 0) transition (after correcting for laser power), and γ(*v*′) is the relative exoelectron efficiency of the *ã* ^3^A_2_ (*v*′) level. The relative exoelectron efficiencies may thus be obtained by dividing the power-corrected intensities by the respective Franck–Condon factors. The Franck–Condon factors for H_2_CO were extracted from the electron energy loss spectrum reported by Taylor et al.^55^ (see Figure S2 of the Supporting Information). No such spectrum is available for D_2_CO, so its Franck–Condon factors were calculated using reduced one-dimensional spectroscopic potentials for modes ν_2_ and ν_4_. The parameters for the ground *X̃* ^1^A_1_ state were obtained from the harmonic force field of Duncan and Mallinson,^56^ whereas the geometry and potential of the *ã* ^3^A_2_ excited state were taken from Jones and Coon.^45^ (See the Supporting Information for details.) The relative exoelectron efficiencies obtained for H_2_CO and D_2_CO after correcting for the Franck–Condon factors are shown in Figure 3b,d, respectively.

## Discussion

Significant differences in exoelectron efficiencies are observed depending on which vibrational mode is excited (Figure 3b,d). In particular, excitation of two quanta of ν_4_ (out-of-plane bending) leads to an increase in exoelectron efficiency relative to the vibrationless *ã* ^3^A_2_ (4^0^) level followed by a slight drop when four quanta of ν_4_ are excited. On the other hand, excitation of one quantum of ν_2_ (CO stretch) leads to a decrease in exoelectron efficiency. These trends are qualitatively similar for both H_2_CO and D_2_CO. In particular, note that the 4^4^ and 2^1^ levels in H_2_CO are nearly isoenergetic (they are separated by only 108 cm^–1^), but they differ significantly in exoelectron efficiency by a factor of γ(2^1^)/γ(4^4^) = 0.30 ± 0.06. We chose to derive the H_2_CO Franck–Condon factors from the experiment of Taylor et al.^55^ (Figure S2). If we instead use calculated Franck–Condon factors obtained from spectroscopically derived potentials (as described in detail in the SI) or calculated *ab initio* values from the literature,^57^ the ratio changes to γ(2^1^)/γ(4^4^) = 0.12 or 0.13, respectively. Any uncertainty that exists in the Franck–Condon factors is insufficient to describe the observed intensity ratios. The exoelectron mechanism seems clearly to depend not on the available vibrational energy above the *ã* ^3^A_2_ origin but on the mode character of the vibration that is excited.

**Figure 4:**
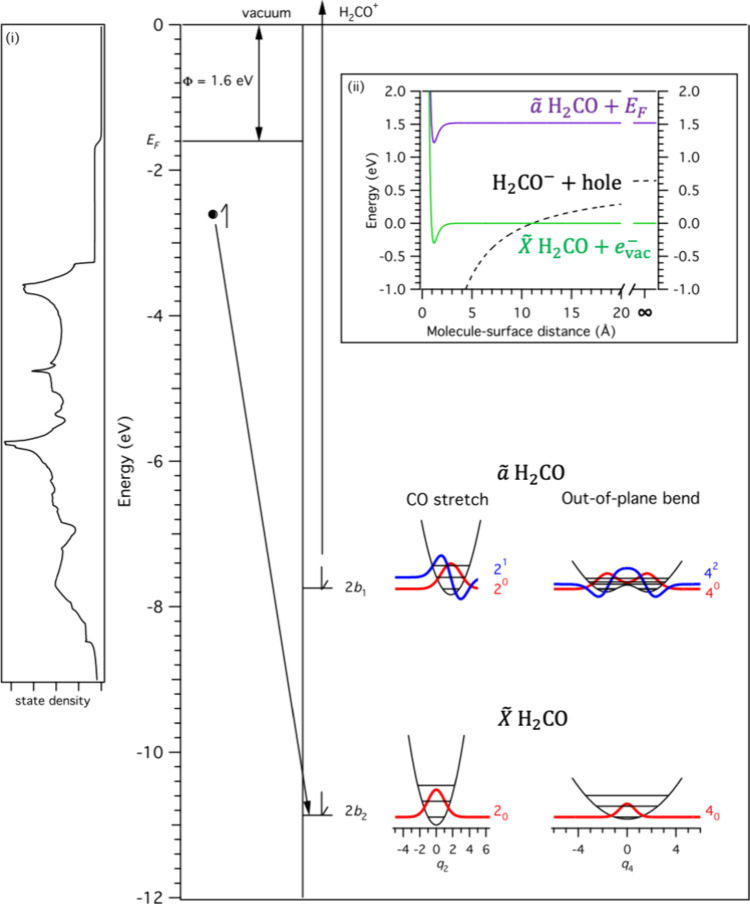
Energetic diagram illustrating the mechanism for Auger de-excitation, in which an electron from the metal surface fills the 2*b*_2_ hole, and the energy released is used to promote the 2*b*_1_ electron to the vacuum. The energies are referenced to the vacuum level of the electron. The left side of the figure shows the energetics of the Cs/Au surface. The Fermi level lies below the vacuum level by an amount equal to the work function (Φ = 1.6 eV). The calculated surface density of states function used in the model is shown in inset (i). The right-hand portion of the figure shows molecular energy levels. The *ã*—*X̃* state separation is 3.12 eV and the *X̃* state lies below the vacuum level by an amount equal to the ionization energy (10.87 eV). Inset (ii) shows the energetics as a function of molecule–surface distance. The neutral *ã* and *X̃* states are modeled using a Morse potential with a physisorption energy of 0.3 eV, whereas the CH_2_O^–^ anion is assumed to take the form of an image-charge potential. *ã* → *X̃* relaxation accompanied by emission of an exoelectron is energetically feasible at any surface distance, and formation of a stable anion is expected to occur only at molecule–surface distances shorter than ∼11 Å. At large surface-distance separations, the energies approach their asymptotic values. The CH_2_O^–^ anion energy lies above the neutral *X̃* state by the negative of the electron affinity (0.65 eV) and the separation between the initial state (*ã* H_2_CO plus an electron at the Fermi level) and the final state (*X̃* H_2_CO plus an electron at the vacuum level) is equal to the *ã*—*X̃* separation minus the work function (3.12–1.6 = 1.52 eV). The bottom right of the figure displays one-dimensional vibrational potentials along the dimensionless generalized normal coordinates, *q*_2_ (CO stretch) and *q*_4_ (out-of-plane bend). Vibrational wave functions for low-lying levels are shown as thick red and blue curves (see text for an explanation of the mechanism for vibrational enhancement or reduction of exoelectron generation efficiency).

The vibrational enhancement of exoelectron efficiency in *a*^3^Π CO scattered from Au(111) has been described in terms of a resonance electron transfer mechanism, in which an electron is transferred at the surface-molecule distance at which the CO^–^ potential energy surface crosses the metastable *a*^3^Π surface.^27,28^ However, such a mechanism is unlikely in the scattering of *ã* ^3^A_2_ formaldehyde from cesium-covered Au(111), because the energetics are different. In the formaldehyde system, the *ã* ^3^A_2_ state lies 0.87 eV *above* the energy of the molecular anion plus surface hole, so that an electron transfer is energetically feasible at any surface-molecule distance (see inset (ii) of Figure 4). Far from the surface, formaldehyde has a negative electron affinity (−0.65 eV), such that spontaneous ejection of the electron into the vacuum is expected in any accessible vibrational configuration. We therefore consider an Auger de-excitation mechanism in which an electron from the metal is transferred to the unoccupied 2*b*_2_ hole of the molecule from which the released energy leads to simultaneous promotion of the 2*b*_1_ electron to the vacuum, as illustrated in Figure 4.

We calculate relative exoelectron efficiencies using a simple model proposed by Zubek,^26^ in which the electron yield for initial and final vibrational states *v*′ and *v*″ is proportional to the integrated density of states that are energetically available for electron emission
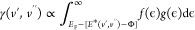
2where *f*(ϵ) is the Fermi function at 300 K, *g*(ϵ) is the density of states in the conduction band, Φ is the surface work function, and E*(*v*′, *v*″) is the total energetic difference (electronic and vibrational) between the initial and final states of the molecule. The surface density of states function, *g*(ϵ), was calculated using density functional theory, as described in the SI. Following the Gurney model,^58,59^ submonolayer coverages of Cs on Au give rise to transfer of the Cs 6s valence electrons to the conduction band of Au. The resulting surface dipole is responsible for the dramatic drop in the work function and brings the Fermi level into partial resonance with the Cs 6s valence band. Consequently, the appearance of the calculated density of states function *g*(ϵ) for the Cs-covered Au surface is similar to that of the Au(111) surface; however, the Fermi level is shifted significantly closer to the vacuum level. The total electron yield from the upper vibrational level *v*′ is obtained by summing over all lower-level contributions weighted by the Franck–Condon factors, *q*(*v*′, *v*″),
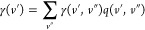
3

We calculated the prediction of this model using one-dimensional Franck–Condon progressions for modes 2 and 4 as described above, assuming that other modes act only as spectators. The results are compared with the experimental observations in Figure 3b,d. The prediction of the model does not quantitatively reproduce the experimental results, but the model correctly predicts some of the qualitative trends. Namely, for both H_2_CO and D_2_CO there is an exoelectron efficiency *enhancement* when the 4^2^ level is excited but a decrease when going from 4^2^ to 4^4^. Although the model correctly predicts a slight *reduction* in efficiency when the 2^1^ level is excited, the magnitude of the reduction is significantly underestimated. Experiments indicate that the exoelectron efficiency drops by about 70% when the molecule is excited from the 2^0^ level to the 2^1^ level of the *ã* state, whereas the model predicts a drop of only 1–2%. The poor quantitative agreement could be due to the neglect of cross anharmonicities or to interactions between the molecule and the surface that might perturb the vibrational potentials of various modes. There may also be inadequacies in the proposed exoelectron ejection mechanism itself. For example, in addition to Franck–Condon effects, vibrational motion along different modes might also distort the molecular orbitals, allowing the 2*b*_2_ orbital to accept an electron more readily. The mechanism could be clarified in more detail by further experiments measuring the exoelectron kinetic energy distributions. However, such experiments are not possible with the experimental setup.

The mode specificity can be qualitatively understood by considering the wave functions shown in Figure 4 for the H_2_CO isotopologue. Although the *ã*–*X̃* displacement along mode 2—which arises because electronic excitation causes the CO bond length to increase from 1.203 to 1.307 Å—gives rise to a short Franck–Condon progression, the 2^0^ level has better overlap with the lowest ground state levels (2_0,1,2_) than the 2^1^ level (see Table S1 of the SI), allowing the 2^0^ level Franck–Condon access to more electrons that are further below the Fermi level. Along the out-of-plane bending coordinate (mode 4), on the other hand, the probability density of the 4^0^ level is maximal at torsion angles close to ±40° due to the out-of-plane double minimum distortion of the *ã* state, so that it does not overlap well with low lying levels of the ground state potential. The 4^2^ level is located just above the inversion barrier, giving rise to a high probability density at the center of the potential, which consequently increases the Franck–Condon overlap with low-lying ground vibrational states, enhancing its access to electrons located further below the Fermi level.

## Outlook

In this work, we have demonstrated clear observations of vibrationally mode-specific energy transfer from molecular vibration to electrons at a low work function surface. The qualitative effects can be understood from a simple model assuming a two-electron Auger process that is governed by Franck–Condon propensities. However, the simple model failed to reproduce the experimental results quantitatively. More detailed future experiments that measure the kinetic energy distributions of electrons ejected from different vibrationally excited states as well as the surface temperature dependence of the exoelectron ejection efficiency will enable a more complete picture of the mechanism to be developed.

## Supporting Information Available

Supporting details about the experimental method and surface dosing conditions; details concerning how Franck–Condon factors were determined, including tabulated results; and description of electronic structure calculations used to derive the surface density of states (PDF)
