# Supplementary material for: Vibrationally Mode-Specific Molecular Energy Transfer to Surface Electrons in Metastable Formaldehyde Scattering from Cesium-Covered Au(111)
Source: J Phys Chem A. 2024 Jun 8;128(25):4976–83. doi: 10.1021/acs.jpca.4c02184 (PMC11215783; doi:10.1021/acs.jpca.4c02184)
Supplement: Supplementary file 1 — jp4c02184_si_001.pdf [file jp4c02184_si_001.pdf]

Supplementary Material for

Vibrationally Mode-Specific Molecular Energy Transfer to Surface Electrons in Metastable Formaldehyde Scattering from Cesium-Covered Au(111)

Behrouz Sabour,[1] Roman J. V. Wagner,[2,3] Bastian C. Krüger,[2,3] Alexander Kandratsenka,[2,3] Alec M. Wodtke,[2,3,4] Tim Schäfer,[2,3] G. Barratt Park[*1,2]

Affiliations

[1]Department of Chemistry and Biochemistry, Texas Tech University, Box 41061 Lubbock, TX, 79409-1061 USA

[2]Max-Planck-Institut für Multidisziplinäre Naturwissenschaften, Am Faßberg 11, 37077 Göttingen, Germany

[3]Georg-August-Universität Göttingen, Institut für physikalische Chemie, Tammanstr. 6, 37077 Göttingen, Germany

[4]International Center for Advanced Studies of Energy Conversion, University of Göttingen, Germany

[*]barratt.park@ttu.edu

Surface Dosing and Stability

While dosing Cs onto the Au(111) surface, the photoemission current that resulted from shining a 633 nm He-Ne laser on the surface was monitored in order to ensure reproducible dosing conditions (Fig. S1). A sharp maximum in the photoemission current is observed after ~4.1 minutes of dosing. This maximum has been observed previously. LaRue *et al.*[1] performed careful measurements of the surface coverage and work function at the peak 633 nm photoemission current. They determined a work function of 1.61 ± 0.08 eV and a surface coverage of 0.22–0.35 ML. When we continue dosing past the initial peak, the photoemission current drops and subsequently rises to reach a plateau after ~7 min. We found that we achieved the most stable surface conditions if we continued dosing until this plateau was reached. Assuming a constant dosing rate, this corresponds to a coverage of 0.38–0.60 ML. The work function of thin layers of Cs on Au has also been investigated by Skottke-Klein *et al.*[2] They found that the system reached a minimum work function of 1.3 eV and increased to an asymptotic value of 1.6 eV at higher coverages. In our work, we assume a surface work function of ~1.6 eV under conditions at which formaldehyde scattering experiments were performed. Although there is some uncertainty about the exact value of the work function, we can confidently set a lower bound of 1.3 eV (the minimum observed in Ref. 2) and an upper bound of 1.96 eV (the He-Ne laser's photon energy) to arrive at a best estimate of $\phi = 1.6 \pm 0.3$ eV. Note in Fig. S1 that the photoemission current under these conditions is stable to within 15% for ~1 h.

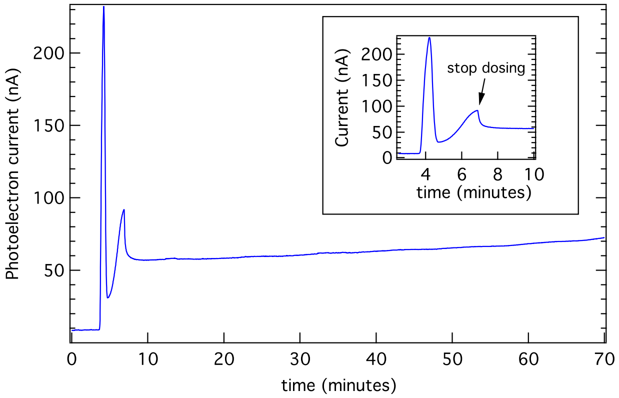

Figure S1. The dosing of a thin layer of Cs on Au(111) is monitored by the photoelectron current induced by shining a 633 nm He-Ne laser on the surface. The sharp peak at ~4.1 min corresponds to a coverage of 0.22–0.35 ML.[1] The photoelectron current subsequently drops and then reaches a plateau after ~7 min, corresponding to a coverage of ~0.38–0.60 ML and an asymptotic work function of 1.6 eV.[2] At this point, we stop dosing. After an initial drop, the photoelectron emission current remains constant (to within 15%) for up to 1 h. All scattering experiments reported in this work were performed within a 30 min window after the dosing is stopped.

One-dimensional Franck-Condon factors between the $\tilde{a}\,^3A_2$ and $\tilde{X}\,^1A_1$ states of formaldehyde

Normal mode coordinates for the $\tilde{X}$ state of formaldehyde were obtained by diagonalizing the **GF** matrix[3] using the parameters of Duncan and Mallinson.[4] The eigenvectors form the columns of the matrix $\mathbf{L}$ that converts internal displacements, $\boldsymbol{S}$, to normal mode coordinates $\boldsymbol{Q}$ according to $\boldsymbol{Q} = \mathbf{L}^{-1}\boldsymbol{S}$. The eigenvectors must be properly scaled to ensure

$$\mathbf{L}^{\dagger}\mathbf{F}\mathbf{L} = \boldsymbol{\Lambda}, \tag{S1}$$

where $\boldsymbol{\Lambda}$ is the diagonal matrix containing the eigenvalues of $\mathbf{GF}$. Dimensionless normalized normal mode coordinates $\boldsymbol{q}$ are obtained using

$$q_i = \left(\frac{4\pi^2\nu_i}{h}\right)^{1/2} Q_i = \gamma_i^{1/2} Q_i, \tag{S2}$$

where ν_i is the frequency of the ith mode. The normal mode displacements between the equilibrium geometries of the $\tilde{a}$ and $\tilde{X}$ states were obtained from

$$\boldsymbol{\delta} = \boldsymbol{\gamma}^{1/2}\mathbf{L}^{-1}\Delta\boldsymbol{S}, \tag{S3}$$

where $\Delta\boldsymbol{S}$ are the internal coordinate displacements.

Franck-Condon factors for the CO stretch mode (ν_2) were obtained in the harmonic approximation. For the out-of-plane bending mode (ν_4) $\tilde{X}$state wavefunctions were obtained using the harmonic approximation, whereas $\tilde{a}$ state wavefunctions were obtained by applying the variational method to the one-dimensional double-minimum potential energy surface of Jones and Coon[5] in the q_4 harmonic basis of the $\tilde{X}$ state. Franck-Condon factors were obtained by direct integration of the wavefunctions. Displacements and harmonic frequencies were obtained from Refs. 4 and 5. The results are tabulated in Tables S1–S4.

Table S1. Calculated one-dimensional Franck-Condon factors for the CO stretch mode (ν_2) of the H_2CO isotopologue. Each row represents an $\tilde{a}$ state vibrational level with quantum number v_2' and each column represents a $\tilde{X}$ state vibrational level with quantum number v_2''.

v_2' / v_2''	0	1	2	3	4	5	6	7	8
0	0.261297	0.400729	0.249082	0.077189	0.011228	0.000429	1.87E-05	2.45E-05	2.40E-06
1	0.294465	0.027837	0.125962	0.31286	0.190655	0.045022	0.002992	3.65E-05	0.000149
2	0.213985	0.034424	0.142395	0.001579	0.216615	0.275877	0.10347	0.011051	8.70E-06
3	0.123372	0.127326	0.015842	0.107231	0.03438	0.089016	0.295663	0.176732	0.028879
4	0.061341	0.15017	0.01758	0.08225	0.02676	0.086261	0.013244	0.251638	0.246961
5	0.027424	0.117114	0.082914	0.006309	0.093521	0.000345	0.088952	0.002126	0.171706
6	0.011306	0.072468	0.114617	0.016072	0.048159	0.051465	0.026785	0.052157	0.026522
7	0.004369	0.038478	0.101627	0.066903	0.001341	0.073814	0.009938	0.060933	0.013758
8	0.001601	0.018281	0.070216	0.094945	0.018394	0.026575	0.060781	0.001099	0.070057

Table S2. Calculated one-dimensional Franck-Condon factors for the CO stretch mode (ν_2) of the D_2CO isotopologue. Each row represents an $\tilde{a}$ state vibrational level with quantum number v_2' and each column represents a $\tilde{X}$ state vibrational level with quantum number v_2''.

v_2' / v_2''	0	1	2	3	4	5	6	7	8
0	0.220519	0.381451	0.274036	0.102531	0.019903	0.001527	1.55E-06	2.43E-05	6.10E-06
1	0.280055	0.053809	0.074632	0.286371	0.224539	0.07166	0.008685	7.75E-05	0.000121
2	0.22335	0.013922	0.152242	0.003732	0.148647	0.282278	0.147495	0.02714	0.00067
3	0.139667	0.100723	0.037193	0.077757	0.068255	0.032284	0.254295	0.224324	0.061318
4	0.074701	0.143749	0.003758	0.098631	0.005954	0.101989	0.000247	0.171567	0.276471
5	0.035714	0.126292	0.058279	0.022376	0.078766	0.011953	0.072175	0.026302	0.082195
6	0.015672	0.085725	0.103938	0.003127	0.067918	0.025023	0.052643	0.023771	0.059234
7	0.006423	0.04924	0.106428	0.044586	0.011894	0.072639	7.87E-05	0.072568	0.000343
8	0.002488	0.025088	0.081708	0.083847	0.004452	0.046339	0.040516	0.014699	0.056971

Table S3. Calculated one-dimensional Franck-Condon factors for the out-of-plane bending mode (ν_4) of the H_2CO isotopologue. Each row represents an $\tilde{a}$ state vibrational level with quantum number v_4' and each column represents a $\tilde{X}$ state vibrational level with quantum number v_4''.

v_4'/v_4''	0	1	2	3	4	5	6	7	8	9	10	11	12	13	14
0	0.214327	0	0.540541	0	0.189543	0	0.042439	0	0.009913	0	0.002414	0	0.000607	0	0.000157
1	0	0.246244	0	0.386295	0	0.23104	0	0.091547	0	0.030874	0	0.00973	0	0.002977	0
2	0.309094	0	0.015077	0	0.192601	0	0.24246	0	0.140804	0	0.0624	0	0.024235	0	0.008754
3	0	0.333528	0	0.031599	0	0.083307	0	0.203563	0	0.171453	0	0.098305	0	0.046409	0
4	0.25323	0	0.063691	0	0.102982	0	0.011092	0	0.131579	0	0.171594	0	0.12854	0	0.073751
5	0	0.236746	0	0.059375	0	0.149804	0	0.004091	0	0.057157	0	0.139644	0	0.142176	0
6	0.137338	0	0.145597	0	0.004323	0	0.151437	0	0.042436	0	0.009088	0	0.08715	0	0.132319
7	0	0.116133	0	0.177706	0	0.005207	0	0.109839	0	0.090814	0	0.002427	0	0.034951	0
8	0.057106	0	0.121845	0	0.106947	0	0.042258	0	0.049786	0	0.115125	0	0.030621	0	0.003832
9	0	0.045401	0	0.167411	0	0.055386	0	0.08891	0	0.007335	0	0.100711	0	0.069922	0
10	0.020123	0	0.06746	0	0.15836	0	0.010777	0	0.114807	0	0.003226	0	0.059184	0	0.093606
11	0	0.015465	0	0.101589	0	0.1436	0	0.001045	0	0.103845	0	0.03256	0	0.017376	0
12	0.006424	0	0.030093	0	0.126773	0	0.101111	0	0.024529	0	0.066779	0	0.070616	0	7.83E-05
13	0	0.004829	0	0.049145	0	0.151971	0	0.051367	0	0.064495	0	0.023906	0	0.091775	0
14	0.001851	0	0.011453	0	0.074631	0	0.161964	0	0.016114	0	0.0981	0	0.002327	0	0.090138

Table S4. Calculated one-dimensional Franck-Condon factors for the out-of-plane bending mode (ν_4) of the D_2CO isotopologue. Each row represents an $\tilde{a}$ state vibrational level with quantum number $v_4{}'$ and each column represents a $\tilde{X}$ state vibrational level with quantum number $v_4{}''$.

$v_4{}'/v_4{}''$	0	1	2	3	4	5	6	7	8	9	10	11	12	13	14
0	0.102324	0	0.503154	0	0.297882	0	0.07299	0	0.017679	0	0.004428	0	0.001136	0	0.000297
1	0	0.15195	0	0.356445	0	0.28518	0	0.132951	0	0.049297	0	0.016528	0	0.005276	0
2	0.235077	0	0.102906	0	0.089927	0	0.243585	0	0.178065	0	0.089756	0	0.037996	0	0.014597
3	0	0.278052	0	0.103571	0	0.021411	0	0.164425	0	0.187668	0	0.126832	0	0.066788	0
4	0.273213	0	0.001879	0	0.147159	0	0.001918	0	0.076395	0	0.158973	0	0.147326	0	0.096744
5	0	0.263704	0	0.00471	0	0.155745	0	0.036754	0	0.016119	0	0.10411	0	0.142288	0
6	0.202037	0	0.075474	0	0.012417	0	0.110297	0	0.086718	0	0.000594	0	0.045846	0	0.111977
7	0	0.16916	0	0.105137	0	0.045437	0	0.052476	0	0.114787	0	0.024684	0	0.007525	0
8	0.110321	0	0.119214	0	0.031474	0	0.089562	0	0.008792	0	0.104148	0	0.064431	0	0.001827
9	0	0.08376	0	0.160955	0	0.007496	0	0.113714	0	0.002232	0	0.063949	0	0.091349	0
10	0.049114	0	0.097477	0	0.110614	0	0.001884	0	0.103542	0	0.02933	0	0.020996	0	0.088931
11	0	0.034973	0	0.132248	0	0.088114	0	0.025433	0	0.065855	0	0.066985	0	0.00033	0
12	0.019184	0	0.058318	0	0.13124	0	0.043713	0	0.06337	0	0.02528	0	0.089885	0	0.010159
13	0	0.013035	0	0.080658	0	0.144959	0	0.010838	0	0.094222	0	0.001474	0	0.083965	0
14	0.006577	0	0.028199	0	0.101344	0	0.133752	0	7.66E-07	0	0.106527	0	0.004591	0	0.063172

The Franck-Condon factors for the $\tilde{a}\ ^3A_2 \leftarrow \tilde{X}\ ^1A_1$ ($2_0^n 4_0^m$) progressions that were used to correct for the relative absorption strengths to obtain the exoelectron efficiencies for the H_2CO isotopologue shown in Fig. 3b of the main text were extracted from the electron energy loss spectrum reported by Taylor *et al.*[6] The spectrum was fitted to a series of Gaussian functions centered at the band center (T_0) of each peak in the Franck-Condon progressions $2_0^n 4_0^m$ (with m even) with a width parameter of $\sigma = 194$ cm^{-1}. Band centers for low-lying vibronic transitions below 27,650 cm^{-1} were taken from Birss *et al.*[7] and the positions of higher lying transitions were adjusted to fit the spectrum (see Fig. S2). No such spectrum is available for D_2CO, so the calculated Franck-Condon factors from Tables S2 and S4 were used to correct for the relative absorption strengths to obtain the exoelectron efficiencies shown in Fig. 3d.

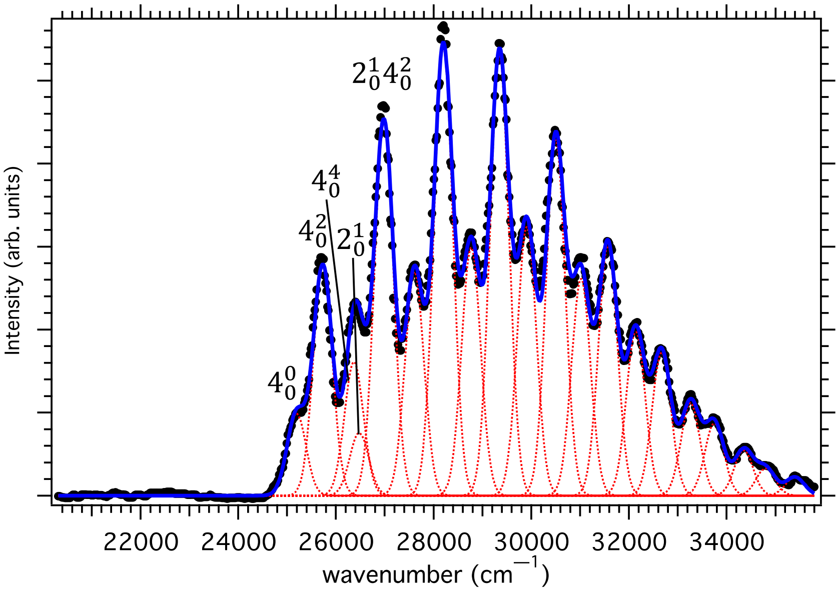

Figure S2. The electron energy loss spectrum of Taylor *et al.*[6] (black circles) was fitted to obtain the relative Franck-Condon factors of the $\tilde{a}\ ^3A_2 \leftarrow \tilde{X}\ ^1A_1$ ($2_0^n 4_0^m$) progressions. Each vibrational band was fitted to a Gaussian (shown by red dotted curves) with a width parameter of $\sigma = 194$ cm^{-1}, located at the band center of each vibronic transition. The band centers of the low-lying transitions below 27,650 cm^{-1} were constrained to the T_0 values reported by Birss et al. and the band centers of higher-lying transitions were adjusted to give the best fit to the spectrum. The summed fit is shown as a solid blue curve.

Density Functional Theory Calculations

The Vienna Ab initio Simulation Package (VASP.5.3.5) was used to perform all periodic density functional theory (DFT) calculations.[8,9] The Perdew-Burke-Ernzerhof (PBE) functional[10] within the generalized gradient approximation (GGA) was used to describe the exchange-correlation interaction. The electron-ion interactions were represented by the projector augmented wave (PAW) method.[11] The Au (111) surface was modeled by a 2x2 5-layered slab, with the top two layers relaxed, separated by a 20 Å vacuum space to avoid the inter-slab interaction. The

geometry optimization was performed using a conjugate-gradient method until the forces acting on each atom were less than 0.01 eV/Å. A Cesium (Cs) atom was positioned at the fcc site on the topmost layer at the distance corresponding to the minimum energy. This geometry results in a surface density of Cs atoms of 0.25 ML. The Brillouin zone integration was performed on a 11×11×1 Monkhorst-Pack k-point mesh. The wave function of the valence electrons was expanded using plane waves with an energy cutoff of 270 eV. Tetrahedron smearing with Blöchl corrections with a width parameter of 0.2 eV was used.

References

1. J. L. LaRue, J. D. White, N. H. Nahler, Z. Liu, Y. Sun, P. A. Pianetta, D. J. Auerbach, A. M. Wodtke, "The work function of submonolayer cesium-covered gold: A photoelectron spectroscopy study" *J. Chem. Phys.* **129**, 024709 (2008), https://doi.org/10.1063/1.2953712.
2. M. Skottke-Klein, A. Böttcher, R. Imbeck, S. Kennou, A. Morgante, G. Ertl, "Preparation and characterization of thin CsAu films" *Thin Solid Films* **203**, 131-145 (1991), https://doi.org/10.1016/0040-6090(91)90524-2.
3. E. B. Wilson, J. C. Decius, P. C. Cross, *Molecular Vibrations: The Theory of Infrared and Raman Vibrational Spectra*. McGraw-Hill, New York, **1955**.
4. J. L. Duncan, P. D. Mallinson, "The general harmonic force field of formaldehyde" *Chem. Phys. Lett.* **23**, 597-599 (1973), https://doi.org/10.1016/0009-2614(73)89037-2.
5. V. T. Jones, J. B. Coon, "Rotational constants and geometrical structure of the $^1A_2$ and $^3A_2$ states of H_2CO and D_2CO" *J. Mol. Spectrosc.* **31**, 137-154 (1969), https://doi.org/10.1016/0022-2852(69)90347-6.
6. S. Taylor, D. G. Wilden, J. Comer, "Electron energy-loss spectroscopy of forbidden transitions to valence and Rydberg states of formaldehyde" *Chem. Phys.* **70**, 291-298 (1982), https://doi.org/10.1016/0301-0104(82)88098-1.
7. F. W. Birss, D. A. Ramsay, S. M. Till, "Further high resolution studies of the system of formaldehyde" *Can. J. Phys.* **56**, 781-785 (1978), https://doi.org/10.1139/p78-103.
8. G. Kresse, J. Furthmüller, "Efficient iterative schemes for ab initio total-energy calculations using a plane-wave basis set" *Phys. Rev. B* **54**, 11169-11186 (1996), 10.1103/PhysRevB.54.11169.
9. G. Kresse, J. Furthmüller, "Efficiency of ab-initio total energy calculations for metals and semiconductors using a plane-wave basis set" *Computational Materials Science* **6**, 15-50 (1996), https://doi.org/10.1016/0927-0256(96)00008-0.
10. J. P. Perdew, K. Burke, M. Ernzerhof, "Generalized Gradient Approximation Made Simple" *Phys. Rev. Lett.* **77**, 3865-3868 (1996), https://doi.org/10.1103/PhysRevLett.77.3865.
11. P. E. Blöchl, "Projector augmented-wave method" *Phys. Rev. B* **50**, 17953-17979 (1994), https://doi.org/10.1103/PhysRevB.50.17953.
